# Enhanced Recovery in Thoracic Surgery: A Review

**DOI:** 10.3389/fmed.2018.00014

**Published:** 2018-02-05

**Authors:** Vesna D. Dinic, Milena Dragisa Stojanovic, Danica Markovic, Vladan Cvetanovic, Anita Zoran Vukovic, Radmilo J. Jankovic

**Affiliations:** ^1^Center for Anesthesiology and Reanimatology, Clinical Center of Niš, Niš, Serbia; ^2^School of Medicine, University of Niš, Niš, Serbia

**Keywords:** enhanced recovery, thoracic surgery, video-assisted thoracoscopic surgery, enhanced recovery after surgery, thoracic anesthesia

## Abstract

The main goal of enhanced recovery program after thoracic surgery is to minimize stress response, reduce postoperative pulmonary complications, and improve patient outcome, which will in addition decrease hospital stay and reduce hospital costs. As minimally invasive technique, video-assisted thoracoscopic surgery represents an important element of enhanced recovery program in thoracic surgery. Anesthetic management during preoperative, intraoperative and postoperative period is essential for the enhanced recovery. In the era of enhanced recovery protocols, non-intubated thoracoscopic procedures present a step forward. This article focuses on the key elements of the enhanced recovery program in thoracic surgery. Having reviewed recent literature, the authors highlight potential procedures and techniques that might be incorporated into the program.

## Introduction

Despite of the advances in surgical and anesthetic techniques as well as improvements in perioperative care, major surgery is still associated with high rate of complications (1, 2). Postoperative complications are associated with prolonged hospitalization, delayed recovery, increased healthcare costs, and poor postoperative quality of life (3, 4). The concept of enhanced recovery after surgery (ERAS), also known as “fast-track,” was derived from the need to minimize hospital length of stay and reduce hospital costs.

Enhanced recovery after surgery program is a multimodal plan of care aimed at optimizing patient before surgery, minimizing patient’s intraoperative stress response, consequently reducing complications, decreasing hospital length of stay and accelerating recovery (5, 6).

**Figure 1:**
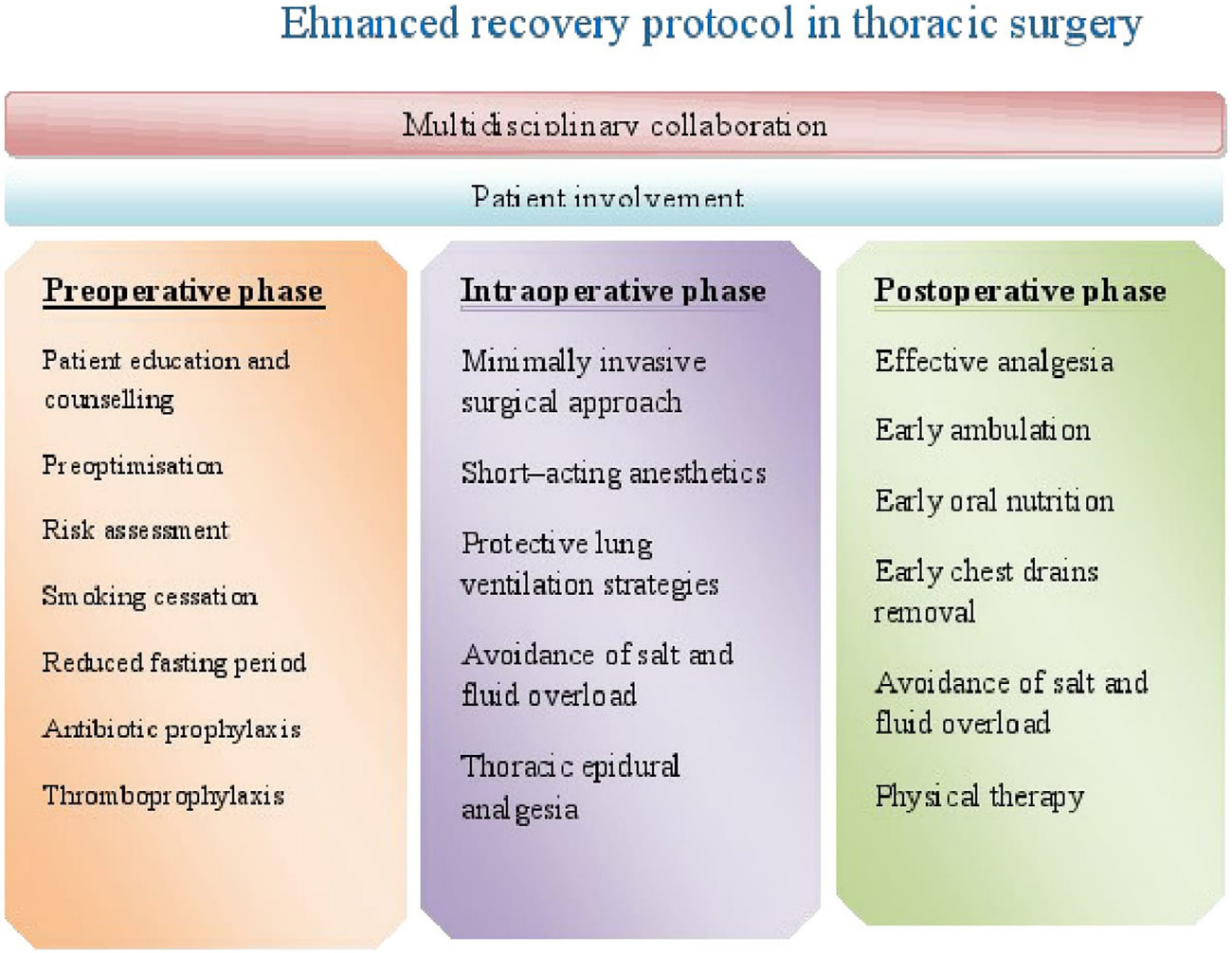
The elements of enhanced recovery protocol in thoracic surgery.

The concept of ERAS was introduced in 1990s by Kehlet (7), and it was primarily intended for patients undergoing elective colorectal surgery (8). Afterward, it has spread to other surgical specialties, showing improvements in terms of clinical outcomes and costs (9). Many of the principles of enhanced recovery in colorectal surgery are adjusted to enhanced recovery protocols (ERP) in thoracic surgery. Although there are variations in care protocols among institutions, the goal of ERP in thoracic surgery is prevention of pulmonary complications as they are the main cause of increased morbidity and mortality in thoracic surgical population (10). The protocol presents an evidence-based approach to patient care which begins in the preoperative period, extends to entire intraoperative period, and ends until hospital discharge. Therefore, it consists of three phases: preoperative, intraoperative, and postoperative (Figure 1).

In this article, we have reviewed the recent literature about ER in thoracic surgery. The PubMed and MEDLINE databases were searched using terms “enhanced recovery,” “thoracic surgery,” “anesthesia,” “fast track,” and “VATS.” Publications from 2000 to 2017 were examined by the authors. To achieve better sensitivity, a search of references of review articles, systematic reviews, and meta-analysis were performed. The search results were limited to English language studies.

## Preoperative Phase

The main goal of preoperative assessment is to identify patients at higher risk, to address modifiable risk factors, and to optimize organ function before the surgery, so the patient could be in the best possible condition for the operation. Therefore, during preoperative phase, attention is focused on the risk assessment and optimization of patient’s medical condition.

Anemia, malnutrition, and chronic obstructive pulmonary disease (COPD) are frequent in patients undergoing thoracic surgery and should be treated before the surgery. Malnutrition is common in cancer patients. It is associated with impaired wound healing, muscle weakness, immune dysfunction, leading to delayed recovery, prolonged length of stay, and costs (11). According to the European Society for Nutrition and Metabolism Guidelines, patients should be screened for malnutrition preoperatively, and those with increased risk should receive nutritional support for 10–14 days before the major surgery (12). Preoperative fasting from midnight is not necessary, as it was proven that patients given fluids 2–3 h preoperatively are not at greater risk of aspiration than those fasted for 12 h (13). Preoperative carbohydrate loading (the night before and 2 h before surgery) is recommended (12). There is evidence that preoperative administration of oral carbohydrate liquids is associated with faster recovery and reduced length of hospital stay (14).

Most of the patients undergoing thoracic surgery are high-risk patients. Preoperative risk assessment is essential for identification of higher-risk patients as they can require more intensive postoperative care and preoptimization. There are still controversies whether these patients should be included in ERAS programme.

Poor preoperative lung function, smoking, and physical inactivity are considered to be the risk factors for complications following thoracic surgery (15, 16).

It has been estimated that active smoking at the time of resection increases the risk of postoperative complications such as pneumonia, myocardial infarction, and stroke. It is also associated with a higher likelihood of death within 30 days after surgery (17). There are still controversies about the optimal time for smoking cessation. One study even reported an increase in perioperative pulmonary complications when smoking cessation occurred just before the surgery (18). Nevertheless, patients should be advised to stop smoking irrespective of timing of operation.

Improving lung function should be one of the main preoperative management strategies. With the aim to improve tolerance to the surgical procedure and enhance postoperative recovery, the concept of preoperative pulmonary rehabilitation was introduced. It integrates exercise training and self-management education. Some studies have shown that preoperative pulmonary rehabilitation can optimize functional state, decrease symptoms, and improve quality of life in patients with COPD (19). The interventions of pulmonary rehabilitation, including exercise training and smoking cessation, were also examined in patients with lung cancer and COPD undergoing lung resection. It has been shown that pulmonary rehabilitation, both before and after surgery significantly improves forced expiratory volume in 1 s (FEV_1_), forced vital capacity, and quality of life (16, 20). Data from a meta-analysis and two systematic reviews have shown that preoperative exercise and smoking cessation in patients undergoing lung resection due to lung cancer, significantly improve pulmonary function and functional capacity, reduce postoperative morbidity and hospital length of stay. However, when exercises were performed only postoperatively, length of stay and postoperative morbidity did not reduce (21–23), which can be explained by the differences in the training programs among the studies.

Available data suggest that preoperative pulmonary rehabilitation, as the part of ERP in thoracic surgery, can improve exercise capacity, as well as reduce postoperative morbidity and mortality in patients with lung cancer. On the other hand, it is recommended not to delay an operation in patients with lung cancer in order to perform preoperative rehabilitation. In the context of aforementioned facts, consensus should be achieved about the training programs, as well as adequate duration of preoperative pulmonary rehabilitation.

Current ERAS guidelines recommend patient education and counseling (24). Patients should be given information about the surgical procedure, anesthesia, and recovery course. They should be encouraged to actively participate in their care as it can contribute to enhanced recovery.

Patient education, preoperative anesthetic assessment, and optimization of patient’s medical condition present an essential part of preoperative phase of ERP in thoracic surgery.

During preoperative phase, special attention should be paid to the airway assessment. One—lung ventilation in patients with difficult airway can be very challenging. Chest radiography and computed tomography are important for airway assessment and selection of an appropriate double-lumen endotracheal tube (DLT). Identification of patients with difficult airway is essential for airway management planning and selection of an appropriate lung isolation device. In patients with already known or anticipated difficult airway the best option to establish an airway is by a single-lumen tube (SLT) while lung isolation can be achieved by bronchial blocker, or an SLT can be substituted with a DLT using an airway catheter technique (25).

## Intraoperative Phase

During intraoperative period, many strategies and techniques can be applied to prevent pulmonary complications.

### Surgical Techniques

The posterolateral thoracotomy (PLT), which is the traditional approach to lung resection, implies muscle-cutting incision. It provides good surgical access, but is associated with increased postoperative pain and reduced respiratory effort (26). With the aim to overcome disadvantages of PLT, muscle—sparing thoracotomy using anterolateral approach and video-assisted thoracoscopic surgery (VATS) were introduced.

Recent studies, including a meta-analysis (27), systematic review (28), and propensity-matched analysis, from Society of Thoracic Surgeons database (29) have shown significantly lower morbidity rate and shorter hospital stay in patients undergoing VATS lobectomy compared with open thoracotomy. The results from these studies are in line with recent findings from the database study from the European Society of Thoracic Surgeon Registry, which compared the outcome following VATS lobectomy versus open lobectomy in case-matched groups of patients (30). The study included 28,771 patients; 26,050 having thoracotomy and 2,721 having thoracoscopy. Compared with thoracotomy, patients undergoing VATS lobectomy had significantly lower incidence of total complications (29.1 vs. 31.7%), wound infection (0.2 vs. 0.6%), and atelectasis requiring bronchoscopy (2.4 vs. 5.5%). Patients undergoing VATS lobectomy had 2 days shorter hospital stay compared with those undergoing thoracotomy, and mortality at hospital discharge was significantly lower in this group (1.0 vs. 1.9%). Concerning patients older than 70 years, the results from this study showed significantly lower number of major cardiopulmonary complications and atelectasis, shorter length of stay, and reduced mortality in the VATS group of patients compared with patients undergoing thoracotomy. The data from these studies confirmed that in comparison to thoracotomy, lobectomies performed *via* VATS are associated with lower incidence of complications, shorter length of stay, and decreased mortality, even in high-risk patients (31). The benefits of VATS on long-term outcomes were also reported. A recent meta-analysis of 20 observational studies reported that compared with open lobectomies, VATS lobectomies were associated with improved long-term outcomes (32).

Due to its beneficial effects on patient recovery, VATS represent one of the main elements of an enhanced recovery in thoracic surgery. To minimize the injury during VATS, the idea of uniportal thoracoscopic surgery has risen. The perioperative outcomes of a single-port, two-port, and three-port approaches were studied. Recent study has shown that VATS single-port and two-port pulmonary resection were associated with decreased volume of drainage, shorter length of stay, and shorter duration of chest drainage (33). However, further randomized controlled trials with larger number of patients are needed to confirm the beneficial effects of single-port compared to three-port VATS pulmonary resections.

### Anesthetic Management

Anesthetic management directed at improving patients’ recovery includes maintenance of normothermia, the use of short-acting agents, protective lung ventilation (PLV), avoidance of fluid overload, and effective analgesia (34). Unlike intravenous anesthetics, volatile anesthetics inhibit hypoxic pulmonary vasoconstriction. However, some data suggest that there is no significant effect of volatile anesthetics on shunt when used in clinically relevant concentrations (35). Recent studies report the suppressive effect of volatile anesthetics and propofol on the alveolar inflammatory response during one-lung ventilation (OLV) (36).

### Perioperative Fluid Management

One of the most severe pulmonary complications in thoracic surgery is an acute lung injury (ALI), presenting the main cause of mortality in patients undergoing lung resection (37). The main task of anesthesiologists is to prevent the development of ALI focusing on the optimal fluid management, by balancing the risks for complications of fluid overload against the risks of hypovolemia and hypoperfusion. Fluid management in thoracic surgery is still controversial topic. There are disadvantages at both sides of regimes—liberal and restrictive. Numerous studies have demonstrated that excessive fluid administration can lead to postresection ALI (38–40).

It has been shown that administration of fluid >2l during pneumonectomy has negative effects on postoperative outcome (41, 42). Similar results are obtained in the studies that evaluated outcome in patients undergoing lesser pulmonary resections managed with high fluid loads (42, 43). On the other hand, there is concern that restrictive regimen can contribute to organ hypoperfusion leading to an acute kidney injury (AKI). In a retrospective study that included 1,442 patients undergoing thoracic surgery with crystalloid restriction <3 ml/kg/h, it was found that the incidence of AKI was 5.1%. The study concluded that fluid restriction neither increased nor was a risk factor for AKI (44). Recent data suggest that crystalloid administration should be <2 l intraoperatively, and <3 l during first 24 h with total fluid balance less than 20 ml/kg during the first 24 h postoperatively (42). Fluid restriction is not just important in prevention of ALI. It has been shown that fluid restrictive therapy leads to earlier resolution of already developed ALI without increasing the risk of AKI (45).

Considering the risk of AKI in a restrictive fluid management, normovolemic and goal-directed therapy protocols were examined. A prospective observational study examined the effects of normovolemia and protective lung ventilation on the development of ALI and found no increase in extravacular lung water (46). However, further studies are needed to find the optimal fluid regimen in patients undergoing pulmonary resections.

### Protective Lung Ventilation

Tissue trauma during surgical intervention, lung hyperinflation, repetitive reexpansion of already collapsed alveoli, and reperfusion during OLV induce cytokine release leading to pulmonary inflammatory response (47).

It has been shown that despite potential hypoxemia, PLV reduces inflammatory response during OLV and consequently the incidence of ALI and postoperative atelectasis (48, 49). The aim of PLV is to minimize pulmonary trauma and avoid respiratory complications including lung injury. It can be achieved by avoiding overdistension and elevated plateau pressure, while providing adequate oxygenation and recruitment of alveoli (50). Although high tidal volumes (10 ml/kg/min) improve oxygenation during OLV, data from animal and human studies suggest that high tidal volumes and high pressures during ventilation are associated with lung injury (43).

Recommendations for OLV suggest that tidal volume of 4–6 ml/kg is protective (50). Protective OLV with low tidal volume is associated with increase in PaCO_2_. Increase of respiratory rate decreases PaCO_2_, but is associated with alveolar colapse–reexpansion cycles leading to atelectotrauma (50). Therefore, permissive hypercapnia is acceptable during protective OLV, while an adequate PEEP applied to the dependent lung keeps the alveoli open, provides oxygenation, and decreases lung injury. In patients with decreased functional residual capacity (FRC), PEEP applied to the dependent lung recruits alveoli and improves oxygenation. However, in patients with increased FRC, PEEP will decrease cardiac output and increase alveolar pressure, which will consequently increase vascular resistance in the dependent lung and increase hypoxemia by diverting blood flow to the non-dependent lung (51).

The value of PEEP should be adjusted according to the respiratory mechanics of the patient, as on the one hand it should prevent lung overdistension, and on the other hand, it should recruit alveoli without hemodynamic impairment.

## Postoperative Phase

During postoperative period, the main goal of ERP is to promote early recovery. Early mobilization, adequate pain control, and postoperative pulmonary rehabilitation present the key elements of the postoperative ERP.

### Pain Management

Pain after thoracic surgery impairs effective chest expansion, coughing, and breathing leading to postoperative atelectasis and pneumonia (52). Therefore, the main goal during postoperative period is to provide effective pain relief as it improves respiratory function and reduces postoperative complications. Regional anesthetic blockade in combination with systemic nonopioid analgesia present the basis of opioid sparing multimodal analgesia in thoracic surgery.

Thoracic epidural analgesia (TEA) is considered the gold standard technique for pain control after thoracic surgery and an essential part of ERAS protocols. In comparison with conventional analgesia techniques, TEA provides superior analgesia for postthoracotomy pain (53), attenuates surgical stress response, having a positive impact on postoperative recovery. However, TEA has several disadvantages including hypotension, urinary retention, and muscular weakness. It can be overcome by performing thoracic paravertebral block (PVB). Recent meta-analysis and systematic reviews confirmed that PVB provides comparable analgesia to the TEA, but with statistically significant lower incidence of side effects, suggesting PVB as analgesic technique for major thoracic surgery (54, 55).

Although negative effects of morphine on respiratory function are described, intravenous patient-controlled analgesia (PCA) with morphine is widely used for pain control following thoracic procedures. To reduce the dose of morphine, ketamin was added to PCA morphine. It was reported that addition of low dose of ketamin to PCA morphine provides better analgesia than PCA morphine alone, reduces morphine consumption, and improves respiratory function (56).

### Chest Drain Management

Chest tubes impair patient mobilization, exacerbate pain, and impose the risk of infection. Early chest tube removal improves forced expiratory volume in 1 s and enhances recovery of vital capacity after thoracic procedures (57). It also reduces pain, allows early mobilization of the patient, and results in shorter hospital stay. Therefore, early chest drain removal represents an important element of fast-tracking protocol in thoracic surgery. Removal of drains is determined by the volume of fluid drainage. Majority of thoracic surgeons prefer leaving the chest tube until fluid drainage decreases to 250 ml/day or less, which prolongs hospital length of stay and delays discharge. Numerous studies have demonstrated that higher threshold for chest drain removal is safe. Nevertheless, there is still debate about the fluid threshold before chest drain removal. Recently, Bjerregaard et al. reported that chest drain removal after VATS lobectomy is safe despite volumes of serous fluid production up to 500 ml/day (58). Unlike these data, data from other studies suggest that 450 ml/day volume threshold for chest tube removal increases the risk for complications (59). However, the consensus on the fluid threshold before tubes removal should be achieved.

## Future Directions

In the era of ERAS protocols, steps forward were made in the field of thoracoscopic surgery. With the aim to avoid complications related to tracheal intubation and to enhance postoperative recovery, efforts have been made to perform VATS procedures without tracheal intubation. The anesthetic technique consists of regional anesthesia and sedation in spontaneously single-lung breathing patient after performing an iatrogenic open pneumothorax (60). The inhibition of cough reflex is achieved by ipsilateral vagal blockade or stellate ganglion block (61, 62). However, open pneumothorax can compromise ventilation and oxygenation in a non-intubated patient leading to hypoxemia and hypercapnia due to carbon dioxide rebreathing from non-dependent lung. Hypercapnia is often mild and well-tolerated, while satisfactory oxygenation is usually maintained *via* facemask (60). Recently, the first reports about the use of transnasal humidified rapid-insufflation ventilatory exchange (THRIVE) in non-intubated VATS have appeared. It has been shown that in comparison to conventional oxygen masks, THRIVE with a flow rate at 20 l/min of oxygen, significantly improves oxygenation during non-intubated VATS, without expanding collapsed lung (63). Growing body of evidence suggests that non-intubated VATS procedures are safe and feasible to various thoracic procedures including pneumothorax management, wedge pulmonary resections, segmentectomy, lobectomy, as well as excision of mediastinal tumors (60). Recent studies reported that in comparison with double-lumen intubated general anesthesia, non-intubated thoracoscopic procedures were superior in terms of complication rate, overall hospital stay, and need for nursing care (62, 64, 65). Data from these studies suggest that non-intubated VATS could become an important element of ERP in the future.

## Conclusion

Enhanced recovery protocol presents an evidence-based approach to patient care. Although there are variations in ERP among the institutions, the evidence suggests that implementation of ERPs in thoracic surgery significantly reduces postoperative complications and length of hospital stay. The role of anesthesiologists is very important during all the three phases of ERP. Minimally invasive surgical technique, adequate perioperative fluid management, protective ventilation, effective pain control, and patient’s active collaboration are essential elements of ERP in thoracic surgery. In the era of fast-tracking, the results of studies regarding non-intubated VATS are promising.

## Author Contributions

All aforementioned authors contributed significantly to the final design of manuscript.

## Conflict of Interest Statement

The authors declare that the research was conducted in the absence of any commercial or financial relationships that could be construed as a potential conflict of interest.
